# P-1351. Occurrence of b-lactamases among Enterobacterales isolates from United States Hospitals in a 10-year period: Report from the International Network for Optimal Resistance Monitoring (INFORM) Program

**DOI:** 10.1093/ofid/ofae631.1528

**Published:** 2025-01-29

**Authors:** Mariana Castanheira, Timothy Doyle, Cory Hubler, Lalitagauri Deshpande, Rodrigo E Mendes, Helio S Sader

**Affiliations:** JMI Laboratories, North Liberty, Iowa; Element Materials Technology/Jones Microbiology Institute, North Liberty, Iowa; Element Materials Technology/Jones Microbiology Institute, North Liberty, Iowa; Element Materials Technology/Jones Microbiology Institute, North Liberty, Iowa; JMI Laboratories, North Liberty, Iowa; JMI Laboratories, North Liberty, Iowa

## Abstract

**Background:**

β-lactamase-production in Enterobacterales challenge the use of β-lactams, the most broadly used and versatile class of antimicrobial agents. The INFORM program surveys β-lactamases and the activity of ceftazidime-avibactam in US hospitals. We analyzed 10 years of data from that study.

Yearly distribution of most common β-lactamases among E. coli (EC) and K. pneumoniae (KPN) isolates collected during 2013-2022 in US hospitals

**Figure:**
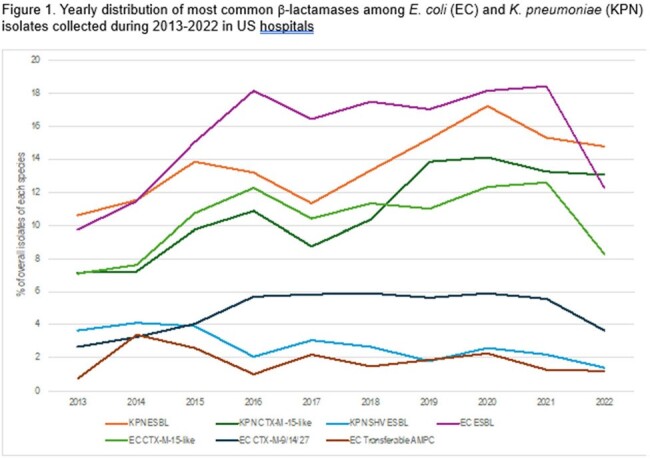

**Methods:**

90,956 Enterobacterales isolates were consecutively collected in 22 US hospitals participating in the INFORM program during 2013 to 2022. Susceptibility testing was performed and interpreted according to CLSI. *E. coli* (EC) and *K. pneumoniae* (KPN) displaying MICs ≥ 2 mg/L for two of: ceftriaxone, ceftazidime, cefepime or aztreonam; or carbapenem-resistant Enterobacterales (CRE; resistant to meropenem or imipenem) were evaluated for the presence of β-lactamase genes. Genetic analysis was performed by microarray/PCR (2013-2015) or whole genome sequencing (2016-2022).

Yearly distribution of CRE isolates collected during 2013-2022 in US hospitals

**Figure:**
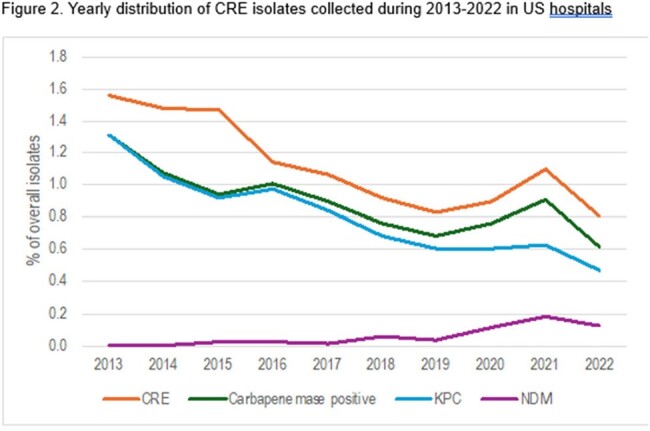

**Results:**

ESBLs were detected among 2,334/2,691 (15.6% of 14,969 overall non-CRE isolates) EC and 964/1,051 (13.8% of 6,988) KPN. A decline in EC displaying the phenotypic criteria and CTX-M-15-like isolates (12.6% in 2021 and 8.3% overall in 2022; Figure 1) was noted in 2022. The most common ESBL in both groups was CTX-M-15-like (10.5/58.3% of the EC overall/characterized and 11.0/73.4% of the KPN). SHV ESBLs had a steady decline among KPN since 2015. CRE isolates declined steadily from 1.6% in 2013 to 0.8% in 2022 (Figure 2). Carbapenemase-positive isolates also declined from 1.3% to 0.6%, with a decline in KPC producers but an increase in isolates producing NDM that were initially detected in 2015 and range from 10-16 isolates in the last 3 study years. Ceftazidime-avibactam and the carbapenems are the most active agents against β-lactamase-producing isolates (Figure 3).

Activity of ceftazidime-avibactam and comparators against main β-lactamase groups

**Figure:**
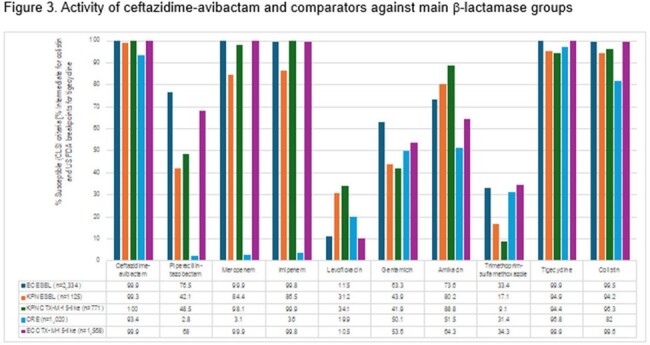

**Conclusion:**

Changes in β-lactamase-producing isolates and its susceptibility profiles should be closely monitored in a local and global level since it impacts patient treatment, but longitudinal data such as this information is scarce.

**Disclosures:**

**Rodrigo E. Mendes, PhD**, GSK: Grant/Research Support

